# Sex and gender differences in cancer pathogenesis and pharmacology

**DOI:** 10.1007/s12094-025-03894-1

**Published:** 2025-04-01

**Authors:** Beatriz Bernardez, Oliver Higuera, Virginia Martinez-Callejo, Ana Cardeña-Gutiérrez, José Antonio Marcos Rodríguez, Ana Santaballa Bertrán, Margarita Majem, Maria-Estela Moreno-Martínez

**Affiliations:** 1 Departament of Medicine and Pharmacology Group, University of Santiago de Compostela, Santiago de Compostela, Spain; 2 Oncology Pharmacy Unit, Pharmacy Service, University Clinic Hospital of Santiago de Compostela, Santiago de Compostela, Spain; 3 Santiago de Compostela Research Institute (IDIS), Santiago de Compostela, Spain; 4 Department of Medical Oncology, La Paz University Hospital, Madrid, Spain; 5 Oncology Pharmacy Unit, Pharmacy Service, Marqués de Valdecilla University Hospital, Avda Marqués de Valdecilla, S/N 39008, Santander, Spain; 6 Department of Medical Oncology, Nuestra Señora de Candelaria University Hospital, Carretera General del Rosario, 145, 38010 Santa Cruz de Tenerife, Spain; 7 Oncology Pharmacy Unit, Pharmacy Service, Virgen Macarena University Hospital, Sevilla, Spain; 8 Department of Medical Oncology, La Fe University Hospital, IISLaFe, Valencia, Spain; 9 Department of Medical Oncology, Santa Creu i Sant Pau Hospital, IIB Sant Pau, Barcelona, Spain; 10 Pharmacy Department, Santa Creu i Sant Pau Hospital, IIB Sant Pau, Barcelona, Spain; 11 Blanquerna School of Health Sciences, University Ramon Llull, Barcelona, Spain

**Keywords:** Cancer, Epidemiology, Gender, Pathogenesis, Pharmacology, Sex

## Abstract

Sex and gender may influence the epidemiology, pathogenesis, and prognosis of cancer. This narrative review describes sex and gender differences in the epidemiology and pathogenesis of cancer, and how such differences may impact the pharmacodynamics and pharmacokinetics of cancer treatment. For most types of cancer unrelated to reproductive function, incidence is higher in males than in females, except for gallbladder and thyroid cancers, which are much more common in women. Cancer mortality is higher in men than women; women account for a larger proportion of survivors. These differences may be related to biological differences in pathogenesis or differences in behaviors relating to cancer risk or detection. The pharmacokinetics and pharmacodynamics of cancer therapies also differ between sexes due to differences in body composition, physiology, and receptor expression. Overall, sex and gender are essential variables to be considered in research and clinical practice, influencing diagnosis, subtyping (biomarkers), prognostication, treatment, and dosage.

## Introduction

Personalized therapy for an individual patient should consider all their characteristics, including their sex and gender. The terms ‘sex’ and ‘gender’ are not the same, although they are often (incorrectly) used interchangeably. Sex refers to the biologically determined features of an individual resulting from chromosomes, reproductive organs, and hormones [1]. Gender is a societal construct based on the expected norms, behaviors, and roles of males and females [2].

There is growing recognition that sex and gender differences in the pathogenesis of cancer have been overlooked, and that scientists have taken a largely ‘nonsexual’ approach to cancer research. For example, according to one analysis, only 2.5% of published articles from The Cancer Genome Atlas (TCGA) have meaningfully addressed sex differences in cancer [3]. Similarly, in an analysis of the 240 biomedical research proposals (in any therapeutic area) approved by the University of Pennsylvania over a 6-month period, only 2% included the intention to examine the impact of sex or gender on primary outcomes [4]. This is despite the fact that the United States National Institutes of Health notes that sex is a biological variable that needs to be factored into the development of research protocols, and recognizes this in their consideration of funding applications [5].

A key issue in cancer research has been the under-representation of women in clinical research, and even female animals or cells in preclinical research. This has led to an incomplete understanding of how sex determines the biological mechanisms that drive cancer in males compared with females. Since gender determines many psychosocial aspects of behavior in health and disease, it is important to also recognize that gender can influence a patient’s risk of cancer and their use of and/or response to treatment. Therefore, in addition to a poor understanding of the impact of sex on disease and treatment, there is an incomplete understanding of how treatment responses (both therapeutic and toxicological) differ between sexes and genders [6–8].

This article reviews the current state of knowledge about sex and gender differences in the epidemiology and pathogenesis of cancer, and how such differences may impact the pharmacodynamics and pharmacokinetics of cancer treatment.

## Methods

A search of PubMed was conducted in November 2023, first using terms for (“sex” OR “gender”) AND (“cancer” OR “oncol*”), and then combining the results of this search with specific terms relating to epidemiology (including frequency, incidence, and prevalence), pathogenesis, pharmacodynamics, and pharmacokinetics. The articles identified were evaluated for relevance and were supplemented by additional articles retrieved from the bibliographies of identified articles, or from ad hoc searches in relation to specific topics.

## Sex disparities in cancer incidence and mortality

GLOBOCAN 2022 data from the International Agency for Research on Cancer indicate that the overall incidence of cancer (all types) is slightly higher in males than in females, with males making up 51.6% and females 48.4% of new diagnoses [9]. However, the incidence rate per 100,000 people is 14% higher in males, with males diagnosed at a rate of 212.5 per 100,000 compared with 186.2 per 100,000 in females [9].

**Fig. 1:**
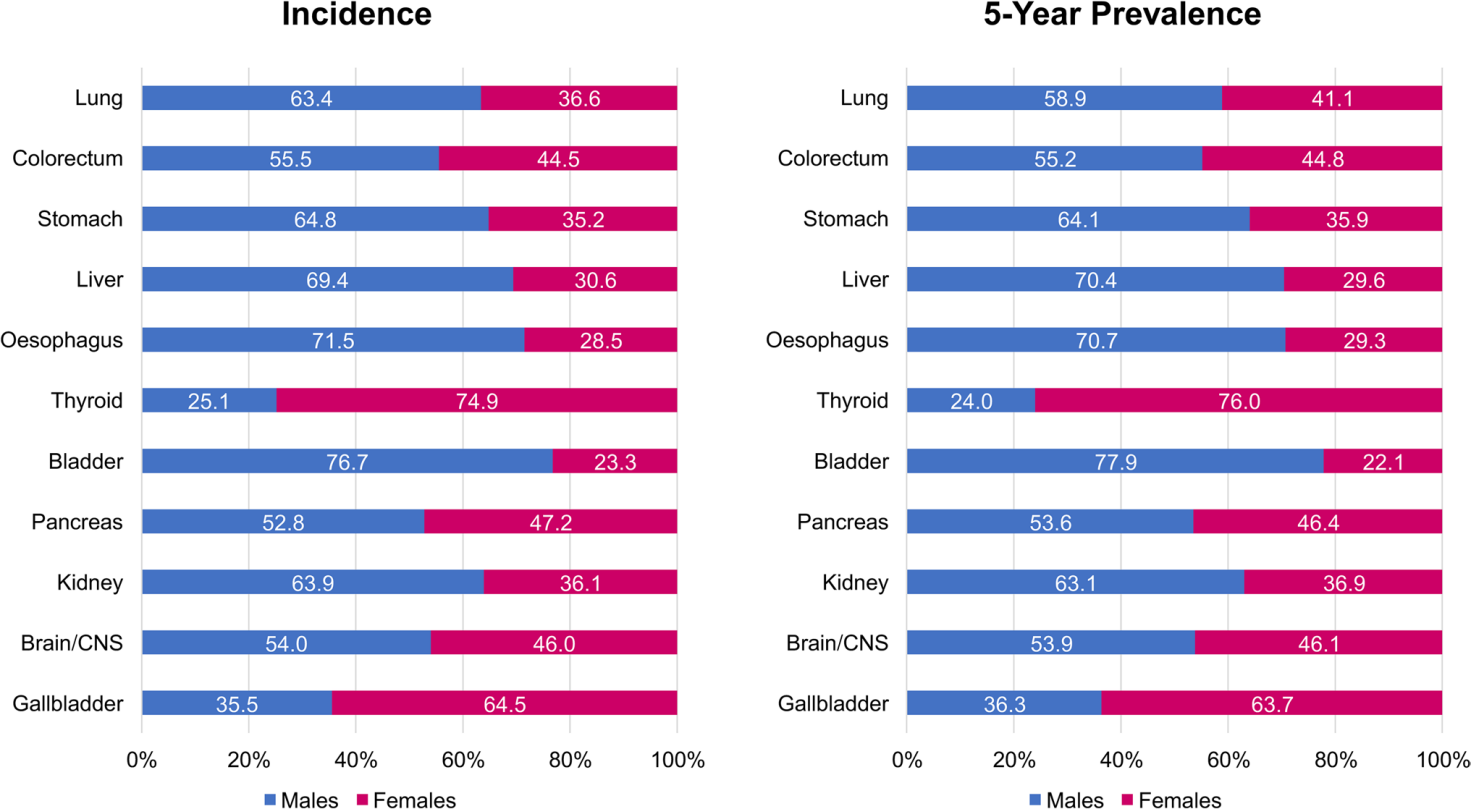
Proportion of males and females among new diagnoses of selected non-reproductive cancers (left) and among those living with selected non-reproductive cancers over 5 years (right) according to GLOBOCAN 2022 data [9, 10]. *CNS* central nervous system

For most types of cancer unrelated to reproductive function, incidence and prevalence are slightly higher in males than in females, with some exceptions. Males account for more than 60% of patients with newly diagnosed cancers of the lung, esophagus, liver, stomach, and kidney, and more than 75% of patients with newly diagnosed bladder cancer (Fig. 1) [9]. On the other hand, females account for almost 65% of patients newly diagnosed with gallbladder cancer and approximately 75% of those with thyroid cancer [9]. Similar sex differences are seen in prevalence rates (Fig. 1) [10].

Similar to incidence, the mortality associated with cancer is generally higher among males than females. Males make up 55.7% of annual cancer deaths, but the mortality rate is 109.7 per 100,000 in males compared with 76.8 per 100,000 in females (a 43% difference) [9]. This leads to more female cancer survivors, and therefore a higher overall 5-year prevalence of cancer among females than males (51.9% vs 48.1%, respectively) [10]. However, this pattern is not consistent across cancer types, with survival generally better in females for melanoma and thyroid, lung, liver, pancreatic, and gastrointestinal tract (colorectal, stomach, esophagus) cancers [11, 12], and better in males for bladder cancer [12] and Kaposi’s sarcoma [11].

## Sex differences in cancer epidemiology

Data show substantial differences between men and women with respect to stage at presentation across a number of cancers, with women generally presenting with disease at an earlier stage compared with men [13–15]. Since stage at diagnosis is a key determinant of cancer-related mortality [16], differences in stage at presentation probably contribute to the lower mortality rate in women than men.

There may be several reasons for this, related to both sex and gender. For some types of cancer, differences in stage at diagnosis may be related to perceptions of risk and, therefore, referral bias by healthcare professionals. For example, men with breast symptoms are less likely to be referred for specialist assessment compared with women with breast symptoms [17]. Another reason may be that women are more likely to engage in self-examination than men, and are, therefore, more likely to identify the visible early signs of cancer such as melanoma lesions or palpable lumps [18].

There are also gender differences in health-seeking behavior. Women are more likely than men to participate in screening programs [18, 19], and in many developed countries, women are much more likely to seek health advice in response to signs and symptoms [20–22]. Delays in seeking medical care among men have been attributed to gender norms of ‘machismo’ or ‘stoicism’, men prioritizing other commitments (e.g., work responsibilities) over their health, and men perceiving health visits as ‘inconvenient’ [23]. On the other hand, many women in developing countries are socio-economically and culturally disempowered, and are, therefore, less likely than men to seek medical advice from healthcare professionals [24, 25].

Sex differences in cancer epidemiology cannot be fully accounted for by differences in health-seeking behavior, nor by differences in diet, tobacco use, alcohol consumption, and environmental exposure [26]. This suggests that underlying pathogenic mechanisms are likely to be important contributors.

## Sex differences in cancer pathogenesis

### X and Y chromosomes in cancer development

The most obvious genetic difference between males and females is the XY and XX dimorphism in sex chromosomes. Females cannot function if both X chromosomes are expressed in their entirety, so X chromosome inactivation (XCI) occurs from the early phase of female embryonic development [27]. Most genes on the inactive X chromosome (Xi) are silenced via C-inactive specific transcript (XIST), a long piece of non-coding RNA that prevents the transcription of one (randomly selected) X chromosome in each pair in most somatic cells [27].

Some of the genes that escape inactivation are tumor suppressor genes, including *MAGEC3, KDM6A, KDM5C, DDX3X, CNKRS2,* and *ATRX*, and these are called escape from X-inactivation tumor suppressors (EXITS) [28]. The presence of two expressed copies of these genes confers females with greater protection against cancer development compared with one copy in males [28]. Several EXITS, including *KDM6A, DDX3X,* and *ATRX*, regulate p53 functions and may help to explain the high occurrence of p53 mutations in male cancers [29].

As a result of XCI, there is considerable mosaicism in gene expression from the maternal or paternal X chromosome, even between adjacent cells, and the potential for skewing if a specific mosaic subpopulation preferentially expresses one X chromosome [27]. Therefore, some of the sex-related differences in cancer epidemiology may be due to the larger number of potential phenotypes in females versus males arising from the distribution of X-linked polymorphic alleles (including for EXITS), and the number of potential genotype combinations possible in females as a result of XCI and X-linked cellular mosaicism [30]. Research suggests that, in addition to mediating XCI, XIST may also directly promote cancer development and growth by suppressing the activity of tumor suppressing microRNAs [31].

Males may also be affected by reduced expression of genes on the Y chromosome, which is termed loss of Y (LOY) for complete loss and extreme downregulation of Y (EDY) for almost complete loss [27]. The presence of LOY is associated with increased tumor burden and genomic instability [32]. In vitro, LOY tumor cells show more aggressive growth and promote more marked T-cell dysfunction and exhaustion, compared with Y-positive tumor cells [33]. EDY is also associated with increased cancer risk in males, even after adjustment for LOY, and is related to *EGFR* overexpression [34].

### Male and female hormones

Sex hormones also likely play a role in the differential incidence of specific cancers in males and females, even in cancers unrelated to reproductive organs [35]. Oestradiol reduces tumor cell proliferation and increases autophagy in many cancers that are more prevalent in males, including esophageal, gastric, hepatic, colorectal, and renal cancer [35]. For example, oestradiol appears to be protective against colorectal cancer (CRC) development [36], which may explain why hormone replacement therapy is associated with a reduced risk of CRC development and improved CRC-specific survival [37].

In non-small cell lung cancer (NSCLC), oestradiol activates the epidermal growth factor receptor (EGFR) pathway and stimulates CXCR4 expression, which promote proliferation, angiogenesis, cell migration, and metastasis [38]. Estrogen receptor (ER) β numbers are increased in NSCLC. These receptors affect mitogen-activated protein kinase (MAPK1) signaling pathways and interact with the heat shock proteins [39].

Oestradiol also increases proliferation in thyroid cancer, which has markedly higher prevalence in females [35]. Similarly, testosterone increases tumor cell proliferation in esophageal, renal, and bladder cancers [35].

**Fig. 2:**
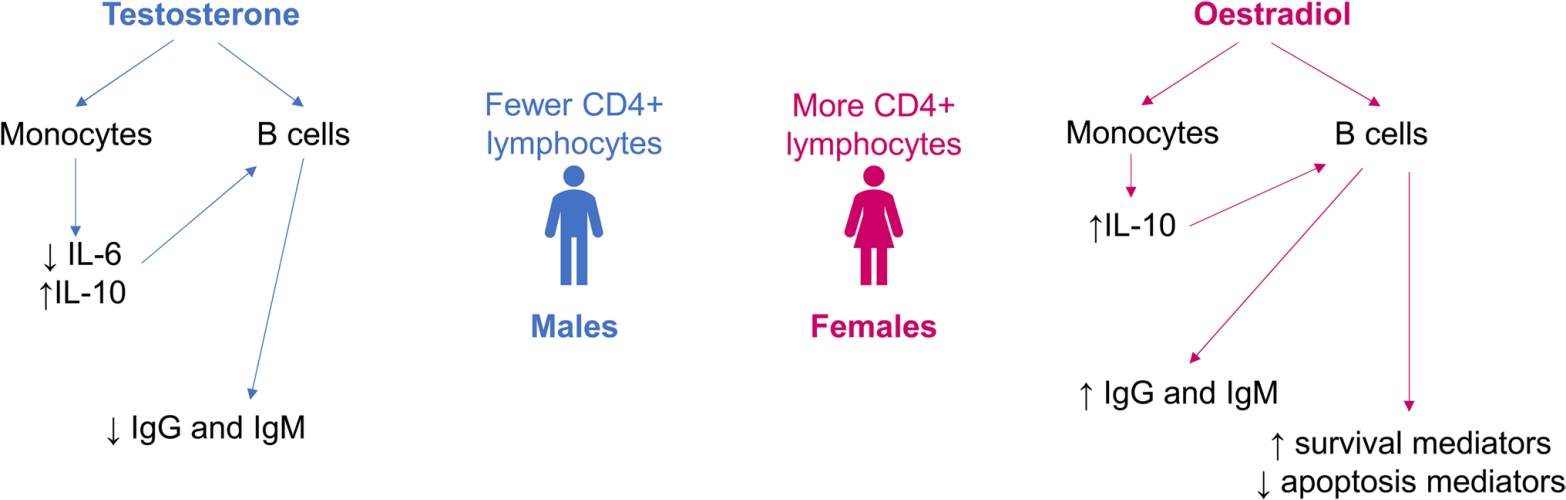
Differential effects of sex hormones on immune cells [40]. *Ig* immunoglobulin, *IL* interleukin. Figure adapted from Irelli A, et al. Biomedicines 2020;8(7):232 (https://doi.org/10.3390/biomedicines8070232), under the terms and conditions of the Creative Commons Attribution 4.0 International (CC BY 4.0) license (http://creativecommons.org/licenses/by/4.0/). Adaptations from the original figure include a simpler presentation of key concepts and a shorter figure legend

Sex hormones have an important impact on the immune system, and this likely accounts for some of the differential behavior of tumors in males and females [40]. Testosterone reduces immunoglobulin (Ig) G and IgM levels directly through its action on B cells, and indirectly by reducing interleukin (IL)−6 production and increasing IL-10 production by monocytes (Fig. 2) [40]. In comparison, oestradiol increases the production of survival mediators (SHP-1, CD22, and Bcl-2) and reduces the production of apoptosis mediators including programmed cell death protein 1 (PD-1) by B cells, and stimulates monocytes to produce IL-10, which in turn increases B cell IgG and IgM production [40].

Estrogens also affect T-cell production and differentiation, neutrophil chemotaxis, and natural killer cell cytotoxicity, depending on their concentration [40]. For example, when estrogen levels are high, there are increased numbers of regulatory T cells (Tregs), greater T-helper (Th) 2 cell differentiation, and reduced secretion of IL-1β, IL-6, and tumor necrosis factor (TNF) by monocytes. Under conditions of low estrogen, Treg numbers are reduced, there is greater Th1 differentiation, and increased secretion of IL-1β, IL-6, and TNF by monocytes [40].

### Genetic and epigenetic differences

Multi-omic data have led to new insights into sex-biased biological processes in cancer, including mutations, epigenetics, and gene regulation [41]. For instance, TCGA datasets have showed significant differences in the number and type of somatic mutations between tumors from males and females [42–44]. Single nucleotide variant numbers (mutation burden) were significantly higher in male versus female urothelial cancers, melanoma, renal papillary cell cancer, and hepatocellular cancer, whereas female glioblastoma showed higher mutation burden compared with male glioblastoma [42]. Mismatch repair genes were affected in some cancers that had a higher mutational burden in males than females, suggesting that the burden was driven partly by sex differences in DNA repair efficiency [42].

Data from the TCGA and other sources show that, within individual tumor types, there are marked differences in driver genes between the sexes, as well as differences in the impact of clinically relevant mutations on outcomes, indicating that the prognostic relevance of genetic markers should be considered separately for males and females [41]. For example, *BRAF* mutation in CRC is associated with poor cancer-specific survival in men but not in women, whereas *KRAS* mutation on codon 13 is prognostic of poor survival in women but not in men [45]. In the TCGA genome-wide analysis of somatic mutations, sex-specific molecular patterns were present for 53% of clinically relevant genes (i.e., those that were a therapeutic target or had prognostic significance) [43].

There are also key differences between the sexes in epigenetic processes, particularly DNA methylation [46, 47], which plays a key role in carcinogenesis and immune signaling [48, 49]. When the methylation of tumor suppressor genes (commonly *RASSF1* and *MGMT*) is dysregulated, apoptosis and DNA repair are affected, leading to tumor growth and resistance to alkylating agents [48]. In vitro tests for methylation markers are being developed as biomarkers for cancer detection and diagnosis [49]; however, patterns of methylation may differ between males and females in specific solid tumor types (Table 1) [50–63]. Currently, none of the genes that show differential methylation between males and females are included in the in vitro diagnostic tests in development [49], but manufacturers should be aware of such differences and account for them when developing biomarker diagnostics for clinical use.

**Table 1:** Sex differences in gene methylation status for specific cancer types

Cancer type	Greater hypermethylation in females vs males	Greater hypermethylation in males vs females
Breast cancer		*RASSF1* [59]
Colorectal cancer	*p14ARF* [57], *p16*^*INK4α*^ [62], *RASSF1* [50]	
Gastric cancer	*GSTP1* [53], *hMLH1* [53], *MGMT* [53]	*CDH1*^a^ [54], *DAPK*^a^ [54], *HACE1* [94], *HOXA11* [51], *THBS1*^a^ [54], *TIMP3*^a^ [54]
Hepatocellular carcinoma	*p16*^*INK4α*^ [56]	*p16*^*INK4α*^ [61]
Non-small cell lung cancer	*CDH13* [58], *GATA5* [58], *KCNH5* [52], *KCNH8* [52], *PAX6* [58], *RARB* [52]	*ERα* [55], *MGMT* [63], *RASSF1* [60]

^a^Chronic gastritis cells

Methylation is not the only epigenetic process that differs between sexes. Chromatin accessibility also varies, and this affects the expression of oncogenes and tumor suppressor genes, including *BRCA1*, *KRAS*, and *MYC*, as well as genes that regulate the immune system, such as *ROS1* [64].

Sex differences in gene expression are mediated by non-coding RNA, including microRNA (miRNA) [41]. The transcription of miRNA differs markedly between sexes, and is directly and indirectly affected by sex hormone receptors [41]. In addition, the X chromosome encodes a much higher number of miRNAs than does the Y chromosome [65]. miRNAs have an important role in immunosurveillance, such as regulating the expression of programmed cell death ligand 1 (PD-L1), either directly or through control of the hypoxia-inducible factor-1α (HIFα) and signal transducer and action of transcription 3 (STAT3) pathways [66]. Some miRNAs are also involved in regulating genes involved in hormone-related signaling pathways, including the PI3K/Akt/mTOR and MAPK/ERK pathways [67]. Sex-related differences in miRNA dysregulation appear to account for at least some of the differences in the epidemiology, presentation, and behavior of specific tumors [65, 66].

### Metabolic and oxidative differences

**Fig. 3:**
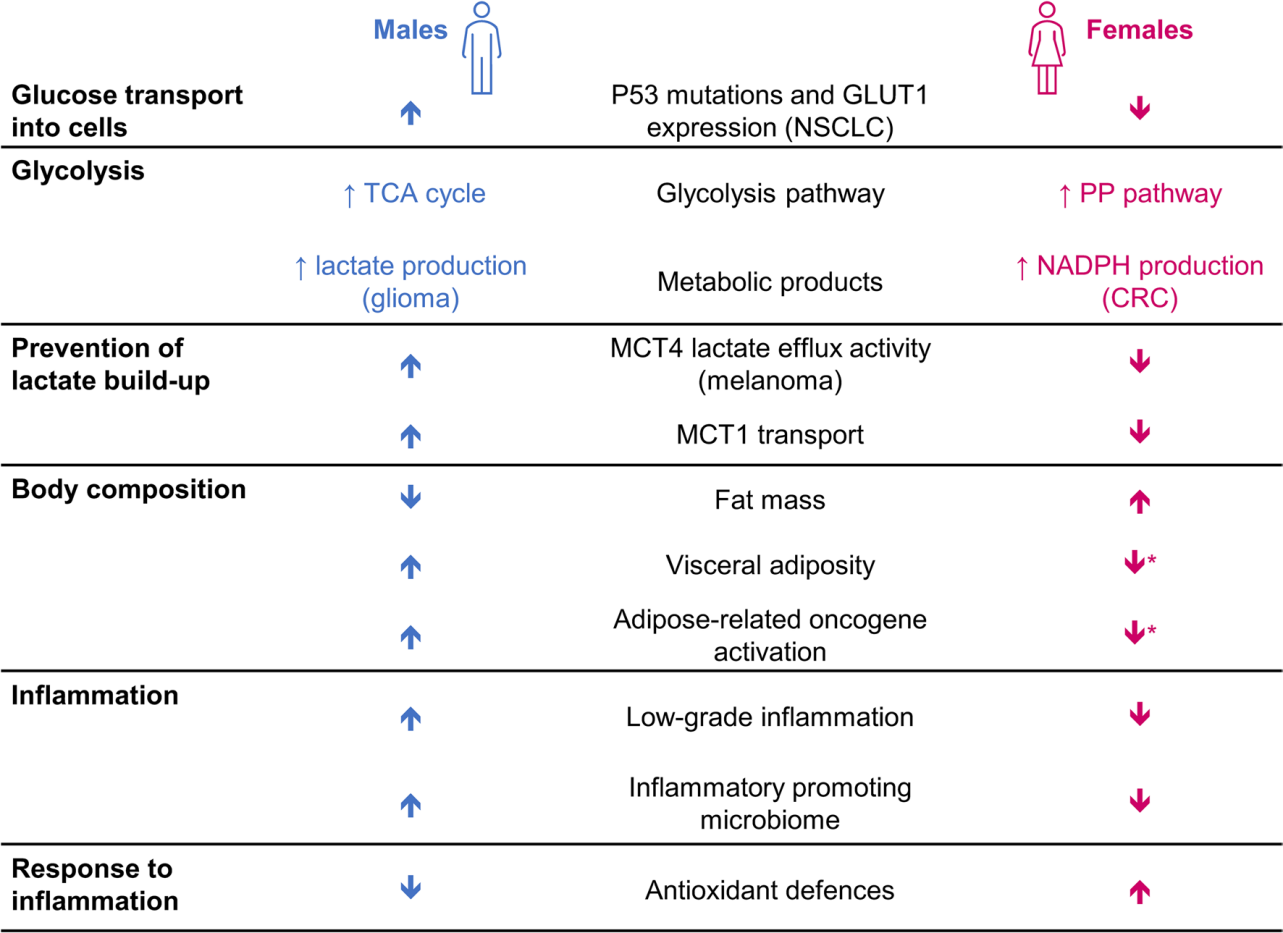
Metabolic differences between male and female cancer cells [27]. *Prior to menopause. *CRC* colorectal cancer, *GLUT1* uniporter glucose transporter 1, *MCT* monocarboxylic acid transporter, *NADPH* nicotinamide adenine dinucleotide phosphate, *NSCLC* non-small cell lung cancer, *PP* pentose phosphate, *TCA* tricarboxylic acid

Metabolic differences between sexes are another potential reason for differences in cancer prevalence and behavior (Fig. 3) [27]. For example, men with NSCLC have significantly greater expression of the glucose transporter GLUT1, which is associated with poor prognosis, compared with women [68]. This transporter is the rate-limiting determinant of glucose uptake, so increased expression of GLUT1 helps to drive cell proliferation [27]. In male cancer cells, the glycolytic pathway preferentially produces lactate via the Warburg effect [69], whereas this is less marked in female cells, which often metabolize glucose via the less energy-efficient pentose phosphate pathway [27]. Increased lactate production can promote epithelial–mesenchymal transition, angiogenesis, and immune evasion [70]. Among male patients with glioma, a metabolomic gene profile associated with high glycolytic activity was associated with poor prognosis, where the same profile was protective in females [71].

There are also sex-related differences in adipose tissue distribution and lipid metabolism, which have been linked to differential cancer risks [27]. Chronic low-grade inflammation associated with visceral adiposity increases the risk of cancer [27], and may explain the increased risk of some cancers after menopause, as fat distribution changes in females [72]. Adipose tissue can promote a proinflammatory cascade either through an extrinsic pathway, in which adipose cell death stimulates inflammatory cytokine release, or via an intrinsic pathway, in which the hyperinsulinemic, hyperglycemic, and hyperlipidemic state promoted by adipose tissue causes activation of oncogenes such as *RAS*, *MYC*, and *RET* [72]. Insulin is not directly oncogenic, but does mediate intracellular signaling pathways that regulate tumor cell metabolism, cell proliferation, survival, and migration [72].

Males and females are also known to differ in their responses to oxidative and inflammatory stress [73]. Males generally show higher levels of basal inflammation and oxidative markers than females, and females tend to have more efficient antioxidant defense mechanisms [73].

## Sex differences in pharmacology

Sex differences in the pharmacokinetics and pharmacodynamics of anticancer drugs have the potential to influence both treatment efficacy and toxicity, as well as optimal dosing strategies [74].

### Pharmacokinetics

**Fig. 4:**
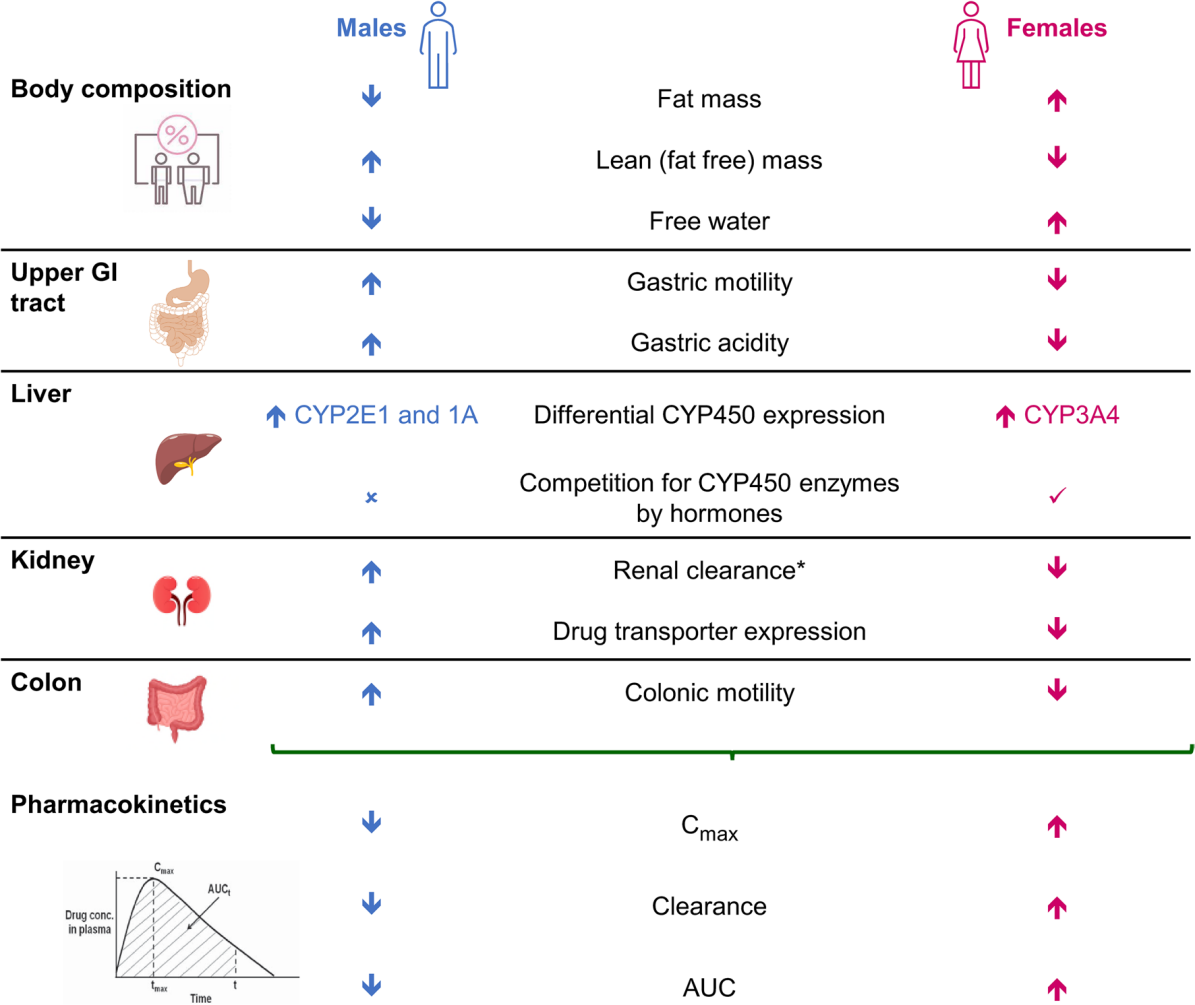
Sex-related anatomical and physiological differences that affect drug pharmacokinetics and generally result in higher systemic exposure in females than in males [2, 75]. *Affected by body mass index. *AUC* area under the concentration–time curve, *C*_*max*_ maximal plasma concentration, *CYP450* cytochrome P450, *GI* gastrointestinal, *t*_*max*_ time to maximal plasma concentration

Absorption, drug distribution, metabolism, and elimination differ between sexes, with maximum plasma concentration (C_max_) and systemic exposure (area under the plasma concentration–time curve [AUC]) generally higher in females than in males (Fig. 4) [2, 75]. Absorption may be differentially affected by the relatively higher gastric pH (lower acidity) and slower gastric transit time in females [75].

Differences in body composition between sexes (i.e., fat-free mass, fat mass, total water content) have a major impact on volume of distribution (Vd). The Vd of water-soluble drugs is higher in males than females but the opposite is true for lipid-soluble drugs [2]. Data show that lower fat-free mass or skeletal muscle mass is associated with an increased risk of chemotherapy-associated toxicity, suggesting that fat-free mass should be considered as a dose parameter [76]. Vd can also be influenced by sex differences in plasma protein binding, which is affected by levels of endogenous and exogenous estrogens [2]. Cardiac output is higher in males than females, meaning that absorbed drug reaches the circulation (including the portal circulation) more quickly in the former [75].

Hepatic metabolism is mainly determined by cytochrome P450 (CYP) enzymes, and the activity of these differs between the sexes. Females show higher levels of CYP3A, whereas males have higher levels of CYP2E1 and CYP1A activity [75]. Estrogens are also metabolized by CYP enzymes; thus, circulating estrogens can compete with drugs for CYP enzymes and affect drug metabolism [75], and exogenous hormone therapy can inhibit or induce CYP enzymes [2].

Renal drug elimination is determined by tubular section, reabsorption, and glomerular filtration rate, and is also influenced by body weight. These variables tend to be higher in males than females; therefore, the clearance of drugs via the renal route is often slower in females than in males [2, 75]. Similarly, fecal elimination of drugs may be more rapid in males than females, because males have faster colonic transit time and higher stool weights, as well as more bile acid secretion and less fiber fermentation [2].

As a result of these factors, females tend to have greater systemic exposure to treatments, which can affect both the efficacy and toxicity of chemotherapy [2]. Pharmacokinetic differences are strongly predictive of sex-specific adverse events during treatment and suggest that dosing should be individualized by sex to prevent overmedication in females [77].

Differences in pharmacokinetic profiles between sexes have been noted for several agents used in the treatment of cancer. A recent systematic review found evidence of significant sex differences in pharmacokinetic parameters for 15 approved anticancer agents, including 8 chemotherapies (5-fluorouracil, carboplatin, doxorubicin, epirubicin, paclitaxel, pegylated liposomal doxorubicin, temozolomide, topotecan), 6 targeted therapies (axitinib, cabozantinib, imatinib, regorafenib, sunitinib, panitumumab), and 1 immunotherapy (atezolizumab) [78]. Potentially significant pharmacokinetic differences were identified for a further eight drugs (docetaxel, irinotecan, oxaliplatin, pemetrexed, raltitrexed, everolimus, trametinib, nivolumab) [78]. Overall, these findings highlight the potential for future sex-based dosing strategies to optimize the risk–benefit ratio of anticancer therapy for both males and females.

More specifically, the 5-fluoropyrimidines (e.g., capecitabine and 5-fluorouracil) show marked differences between the sexes, with lower elimination and higher adverse event incidence in females versus males [79, 80]. The elimination of 5-fluoropyrimidines is influenced by body composition, and it has been suggested that dosages should be calculated in relation to fat-free mass rather than body surface area (BSA) or body weight [74].

Conversely, doxorubicin clearance is higher in men than women, with one study showing a correlation between doxorubicin clearance and height but not weight or BSA [81]. The same is true for pegylated liposomal doxorubicin (PLD), which is cleared primarily by the mononuclear phagocytic system [82, 83]. In this instance, the sex difference in PLD clearance is attributed to the effects of estrogen and testosterone on the immune system [82].

Paclitaxel pharmacokinetics are also influenced by sex. Female patients have higher C_max_ compared with male patients, as well as significantly greater time above the paclitaxel threshold concentration (0.1 μmol/L), making females more vulnerable to hematotoxicity [84]. Although the mechanism is unclear, male hormones affect taxane pharmacokinetics, with higher clearance seen among castrated versus non-castrated men with prostate cancer [85].

Topoisomerase inhibitors also show different pharmacokinetic profiles between males and females. The C_max_ and AUC of irinotecan correlate with BSA and body mass index, and are higher in men than women [86]. The clearance of topotecan is lower in women than in men; this difference is primarily related to lower hematocrit in female patients, since topotecan is carried by erythrocytes [87].

### Pharmacodynamics

Relative to pharmacokinetics, less is known about pharmacodynamic differences between the sexes. However, sex hormones can affect hepatic enzyme activity [75], and sex hormone receptors (e.g., ER) can mediate drug resistance by modulating downstream signaling pathways and the expression of efflux proteins from the ATP-binding cassette family, including breast cancer resistance protein [88].

Sex-related differences in immune function (described earlier) likely produce differential effects of immune checkpoint inhibitors (ICIs) between the sexes [2]. Females tend to show a more robust antigen-driven immune response [2] and a lower tumor mutational burden [89, 90]. This may affect the antigenicity of the tumor and therefore the efficacy of ICI therapy [91], with males tending to show greater therapeutic responses to ICIs than females [2]. In addition, genes related to T-cell immune response are overexpressed in females compared with males, causing stronger inflammatory and cytotoxic T-cell responses that may lead to an increased risk of immune-related adverse events (e.g., ICI myocarditis) in females [92]. Furthermore, the X chromosome contains many genes involved in the regulation of immune function (e.g., IL-2 receptor, IL-3 receptor and the toll-like receptor 7); therefore, XCI and the resultant cellular mosaicism in females may also play a role in sex-based differences in ICI efficacy and immune-related adverse events [92].

Sexual dimorphism in the expression of other receptors can also influence drug activity, such as the known difference in the analgesic effect of opioid drugs in males and females. These agents have decreased efficacy in women due to sex differences in μ- and κ-opioid receptor expression and signaling [2]. Females also experience a reduced analgesic response to opioids due to differences in toll-life receptor-4 signaling in the midbrain periaqueductal grey, as well as the influence of sex hormones in this region [93].

## Conclusion

There are multiple potential pathogenic mechanisms for sex differences in cancer epidemiology and behavior, as well as pharmacological mechanisms for sex differences in treatment impact. These differences highlight the need to consider sex as an important variable in cancer research, and to develop sex-specific approaches to cancer diagnosis, subtyping (biomarkers), prognostication, and treatment in clinical practice. Limited data on the influence of gender on cancer epidemiology, pathogenesis, and treatment are available, underscoring the need for further research in this area.

## Data Availability

Data sharing is not applicable to this article as no datasets were generated or analyzed during this work.
